# Why ethics guidance needs to be updated for contemporary HIV prevention research

**DOI:** 10.1002/jia2.25500

**Published:** 2020-05-14

**Authors:** Brandon J Brown, Jeremy Sugarman

**Affiliations:** ^1^ Center for Healthy Communities School of Medicine University of California Riverside CA USA; ^2^ Berman Institute of Bioethics Johns Hopkins University Baltimore MD USA

**Keywords:** HIV prevention, research ethics, guidelines, stakeholders, HIV Prevention Trials Network

Despite existing effective antiretroviral treatments and means of prevention, the human immunodeficiency virus (HIV) epidemic persists globally [1]. While efforts to scale‐up access to these modalities is critical, research is needed to expand the range of options available to curb HIV incidence. Nevertheless, conducting high‐quality HIV prevention research necessitates surmounting a range of obstacles. For instance, much HIV prevention research encounters ethical challenges in practice, particularly in settings with high incidence marked by weak healthcare infrastructures, poverty, laws adversely affecting key populations, inequality, discrimination and/or stigma. In addition, since HIV transmission is a high consequence, but relatively low probability event in the context of research, HIV prevention efficacy trials tend to be very large, sometimes requiring thousands of participants, making them extremely expensive. Furthermore, in order to show that an intervention is generalisable in diverse environments, these trials tend to be multisite and multinational, adding complexity.

As a result of such realities associated with conducting HIV prevention research, major funders have looked to networks and other research consortia, like the HIV Prevention Trials Network (HPTN), to coordinate these trials. Moreover because of the sensitive nature of HIV prevention research being conducted globally, ethics guidance that is universally applicable is needed.

## 1 RECENT DEVELOPMENTS RELEVANT FOR ETHICS GUIDANCE

Practical ethics guidance for contemporary HIV prevention research must not only be sensitive to these issues, but also be responsive to numerous developments. For example, advances in HIV prevention science (e.g. treatment as prevention and oral pre‐exposure prophylaxis) introduce complex ethical issues in the design of HIV prevention trials, such as selecting ethically appropriate comparator arms [2, 3, 4]. Here there are important tensions between the need to protect research participants by providing them with known means of HIV prevention and the ability to implement trials capable of meaningfully evaluating potentially powerful new interventions. In addition, ethicists and others increasingly emphasize the importance of addressing research questions that are relevant to local populations and responsive to host communities’ health priorities [5, 6, 7, 8], but which can complicate conventional approaches to selecting research sites that may have simply relied upon HIV incidence data within a particular locality. While community engagement and capacity building have long been part of much HIV prevention research, many guidance documents articulate specifications for robustly engaging communities and strengthening local capacity beyond health care and performing research [9, 10, 11, 12, 13, 14, 15, 16, 17], making explicit a broad range of responsibilities for HIV prevention researchers that necessitate careful attention.

Several guidelines, policies and regulations have also evolved recently, introducing a range of expectations and requirements for HIV prevention research. The updated Council for International Organisations of Medical Sciences guidelines place greater importance on the social value of research, which ought to be considered when considering whether particular research endeavours are pursued. They also delineate requirements for research among those who might become pregnant during the course of a study, which are essential to consider given the public health and ethical mandates to test HIV prevention modalities in this population [5]. The latest version of the Declaration of Helsinki emphasizes the importance of post‐trial provisions “for all participants who still need an intervention identified as beneficial in the trial,” establishing privacy protections, assessing capability of giving informed consent, and providing study results to participants [18]. All of these issues are relevant to HIV prevention research. Further, the revised US federal regulations for research with human subjects, known as the “Common Rule,” includes provisions related to broad consent and biospecimens (45 CFR 46.116) and encourages research ethics review by a single committee (i.e. Institutional Review Board in the US) (45 CFR 46.114.b.1) [19]. While these possibilities may be welcome and appropriate for some research, they can be problematic in internationally collaborative HIV prevention research that can encounter ethical, cultural and legal barriers in work with biospecimens and where local ethics oversight may be especially important for ensuring the social context is taken into account so that the welfare of participants is protected.

Additionally, standards have changed regarding access to and dissemination of research results, such as providing participants with health‐related results and third party researchers with access to raw datasets for analysis [20, 21, 22, 23]. The HPTN and other stakeholders have also published empirically derived guidance regarding post‐trial access to successful interventions [24, 25]. Finally, molecular epidemiology has been increasingly playing a role in HIV prevention research and raises ethical, legal and social issues related to directionality of HIV transmission, which could result in stigma, discrimination and criminal prosecution [26].

## 2 THE HPTN’S RESPONSE TO CHANGES IN THE CONTEXT OF HIV PREVENTION RESEARCH

The HPTN is a worldwide collaborative research network that includes researchers, community members, ethicists and others that develops and tests the safety and efficacy of interventions designed to prevent the acquisition and transmission of HIV. In 2003, to help ensure that its research was ethically sound, the HPTN developed the *HPTN Ethics Guidance for Research* [27, 28]. The guidance was updated in 2009 [29] and is an important source document regarding the ethical issues in HIV prevention research globally. Although there are other ethics guidance documents for HIV‐related research, the HPTN guidance is intended to offer a practical approach to identifying and addressing ethical issues in the practice of HIV prevention research that is sensitive to the potentially competing claims of policies and other guidance documents.

To provide those engaged in HIV prevention research with practical guidance regarding such ethical issues, the HPTN 2009 ethics guidance document was revised following an extensive process. It was approved and posted on February 26, 2020 [30].

## 3 ETHICS GUIDANCE POINTS AND PRIMARY STAKEHOLDERS

The revised ethics guidance document is organized according to the different stages of HIV prevention research, from research preparation, to implementation, and dissemination. The points are outlined in the Table 1; they are each explicated in the primary document. One major way that the current guidance is unique is that each guidance point identifies the primary stakeholder(s) responsible for implementing each of the ethics guidance points, and also specifies whether each guidance point is an ethical obligation that must be met or is an ethical aspiration, which is desirable but not required. Nevertheless, as described in the HPTN ethics guidance: “in general, all stakeholders in HIV prevention research are encouraged to fulfil their ethical obligations and to pursue ethical aspirations to the greatest extent possible [30, p. 10].”

**Table 1:** Ethics guidance points

Guidance point	Description	Ethical obligation versus aspiration	Who is responsible
1. High‐quality scientific and ethical research	Those engaged in HIV prevention research must be committed to designing and implementing high‐quality scientific research and research ethics practices throughout the research process	Obligation	Sponsors and researchers
2. Research objectives and priorities	HIV prevention research should prioritize efforts that address public health needs, reduce health inequities, and are locally relevant	Obligation	Sponsors and researchers
3. Community engagement	Relevant communities should be actively engaged throughout the research process to help ensure that HIV prevention research is appropriate as well as scientifically and ethically sound	Obligation	Researchers, study teams, and community representatives
4. Local capacity and partnerships	HIV prevention research should seek to develop local capacity and establish collaborative partnerships	Aspiration	Sponsors, researchers, study team and research sites
5. Study design	HIV prevention research should be designed to minimize risks and maximize benefits to study participants and their communities, while remaining scientifically sound	Obligation	Researchers and sponsors
6. Consent, assent, permission and re‐consent	Each site involved in HIV prevention research should develop, implement and document appropriate informed consent, assent, permission and re‐consent processes tailored to the informational needs of participants	Obligation	Researchers and study teams
7. Addressing vulnerabilities	HIV prevention researchers should assess, monitor and respond to the social, cultural and other factors that may place research participants at heightened risk	Obligation	Study team and researchers
8. Ethical review of research	Independent ethics review committees in host countries should review HIV prevention research	Obligation	Sponsor, researchers research sites and ethics review committees
9. Standard of prevention	HIV prevention researchers should partner with key stakeholders to provide a package of effective, comprehensive and sustainable prevention services to all participants in HIV prevention research	Obligation (provision of prevention package) and aspiration (content of prevention package)	Study team and sponsors
10. Standards of care and treatment	HIV prevention researchers should strive to provide care and treatment to participants that exceed local standards of medical services, yet does not impose undue influence to participate in research	Obligation (establishing standards of care and treatment) and aspiration (content of standards)	Researchers and study team
11. Independent data safety and monitoring	HIV prevention researchers and sponsors should ensure that appropriate mechanisms for independent data and safety monitoring are in place	Obligation	Sponsors, researchers and study teams
12. Disseminating research results	HIV prevention researchers should plan for the timely communication of HIV prevention research results to scientific audiences as well as participants, affected communities, and other stakeholders in a manner that promotes understanding and trust	Obligation	Study team, sponsor, researchers, community representatives
13. Sustaining capacity‐strengthening and infrastructure	HIV prevention researchers should endeavour to ensure that the investments made in developing capacity will continue to provide benefits and opportunities for local researchers and communities after research ends	Aspiration	Researchers, sponsors and research sites
14. Continuing care for research participants	HIV prevention researchers should seek to facilitate continuity of prevention services and care for participants who still require it after research participation has ended	Aspiration	Researchers and study team
15. Post‐trial access to effective interventions	HIV prevention research seeking to establish the efficacy of an intervention must have at minimum a preliminary plan regarding post‐trial access to interventions proven to be safe and effective, which offer meaningful benefit for research participants and their communities	Obligation (preliminary plan regarding the provision of successful interventions to participants) and aspiration (provision of successful interventions to participants, communities and at‐risk populations)	Sponsor, researchers, study team and local partners

The current version of the HPTN ethics guidance aims to have wide applications for the HIV prevention research field at large both inside and outside of the HPTN, and have multiple audiences. Researchers, collaborating institutions, community advisory boards, industry sponsors, ethics review committees, and other stakeholders can utilize it to reflect upon, guide or support ethical decision making in HIV prevention research.

HIV prevention research is essential to developing new tools and approaches to address the HIV epidemic by decreasing HIV incidence. By expanding upon the fundamental ethical principles of research and specifying them for practical application in research, the HPTN ethics guidance document is positioned to help ensure that this it is designed and conducted responsibly. This is essential to protecting the rights, interests and welfare of those engaged in and affected by the research. It is expected that as new ethical issues emerge over time, these guidelines will be revisited.

## Competing interests

Brandon Brown has nothing to disclose. Jeremy Sugarman is a member of Merck KGaA’s Bioethics Advisory Panel and Stem Cell Research Oversight Committee; IQVIA’s Ethics Advisory Panel; and has consulted for Portola Pharmaceuticals Inc. None of these relationships are related to the material discussed in this manuscript.

## Authors’ contributions

BB drafted an initial version of this manuscript, made critical revisions to it and approved the final version. JS conceived of the idea of the manuscript, made critical revisions to it and approved the final version.
